# Society Notes

**Published:** 1904-01

**Authors:** 


### Society Notes.

---

THE AUSTIN DISTRICT MEDICAL SOCIETY held its regular annual meeting in Austin December 22, ult. Dr. E. M. Thomas of Georgetown was elected president; Dr. W. A. Harper was elected secretary.

---

THE BRAZOS VALLEY MEDICAL ASSOCIATION held a rattling good meeting at Hearne on the 13th and 14th inst. More anon.

---

CHEROKEE COUNTY MEDICAL SOCIETY.—Dr. Holman Taylor of Marshall, State Medical Association Councillor for Fifteenth District, rounded 'em up at Jacksonville on December 21st, and organized the Cherokee County Medical Society under the new regime. Dr. H. O. Collins of Jacksonville was elected president; Dr. Bingham of Rusk, vice president, Dr. J. B. Ramsay of Alto, secretary. Next meeting, Jacksonville, January 13th, inst.

---

THE SMITH COUNTY MEDICAL SOCIETY met in Tyler, December 21st, ult., and adopted the charter recommended by the State Medical Association. Dr. Irvin Pope of Tyler was elected president; Dr. J. C. Smith, vice president, and Dr. A. Woldert, secretary and treasurer; censors, Drs. T. J. Bell, J. B. Phillips, J. Z. Ferrell. They wound up with a banquet, at which many toasts were drunk, and all went as merry as a marriage bell.

